# Effects of Sirolimus Treatment on Fetal Hemoglobin Production and Response to SARS-CoV-2 Vaccination: A Case Report Study

**DOI:** 10.3390/hematolrep15030044

**Published:** 2023-07-12

**Authors:** Maria Rita Gamberini, Cristina Zuccato, Matteo Zurlo, Lucia Carmela Cosenza, Alessia Finotti, Roberto Gambari

**Affiliations:** 1 Center “Chiara Gemmo and Elio Zago” for the Research on Thalassemia, Università degli Studi di Ferrara, 44121 Ferrara, Italy; 2 Unità Operativa Interdipartimentale di Day Hospital della Talassemia e delle Emoglobinopatie, Arcispedale S. Anna di Ferrara, 44124 Ferrara, Italy; 3 Department of Life Sciences and Biotechnology, University of Ferrara, 44121 Ferrara, Italy

**Keywords:** β-thalassemia, sirolimus, SARS-CoV-2, vaccine, immunity

## Abstract

The β-thalassemias are a group of monogenic hereditary hematological disorders caused by deletions and/or mutations of the β-globin gene, leading to low or absent production of adult hemoglobin (HbA). For β-thalassemia, sirolimus has been under clinical consideration in two trials (NCT03877809 and NCT04247750). A reduced immune response to anti-SARS-CoV-2 vaccination has been reported in organ recipient patients treated with the immunosuppressant sirolimus. Therefore, there was some concern regarding the fact that monotherapy with sirolimus would reduce the antibody response after SARS-CoV-2 vaccination. In the representative clinical case reported in this study, sirolimus treatment induced the expected increase of fetal hemoglobin (HbF) but did not prevent the production of anti-SARS-CoV-2 IgG after vaccination with mRNA-1273 (Moderna). In our opinion, this case report should stimulate further studies on β-thalassemia patients under sirolimus monotherapy in order to confirm the safety (or even the positive effects) of sirolimus with respect to the humoral response to anti-SARS-CoV-2 vaccination. In addition, considering the extensive use of sirolimus for the treatment of other human pathologies (for instance, in organ transplantation, systemic lupus erythematosus, autoimmune cytopenia, and lymphangioleiomyomatosis), this case report study might be of general interest, as large numbers of patients are currently under sirolimus treatment.

## 1. Introduction

The β-thalassemias are a heterogenous group of monogenic hereditary hematological disorders caused by deletions and/or mutations of the β-globin gene [1,2,3]. Since these alterations are associated with a total (β^0^) or a partial (β^+^) suppression of the expression of this gene, causing the absence or reduction of adult hemoglobin (HbA) production, the reactivation of the silent γ-globin genes can ameliorate the clinical parameters of β-thalassemia patients in association with a “de novo” production of fetal hemoglobin (HbF) [4,5,6,7,8].

The mTOR (mammalian target of rapamycin) inhibitor sirolimus [9,10,11] has been proposed as a potent HbF inducer in vitro [12,13] in experimental animals [14,15,16] and when administered to patients carrying sickle-cell disease (SCD) [17,18] and β-thalassemia [19] traits. On the other hand, mTOR inhibitors retain immunomodulating properties [20]. Therefore, especially considering the coronavirus disease 2019 (COVID-19) pandemic [21,22], the use of mTOR inhibitors should be carefully monitored with respect to potential effects on humoral (i.e., production of neutralizing antibodies) [23] and cellular (i.e., memory T-cell) [24,25] responses against severe acute respiratory syndrome coronavirus 2 (SARS-CoV-2) antigens. This is of great relevance for the following considerations: (a) sirolimus is the prototype of a large class of mTOR inhibitor analogues; (b) it was approved by the FDA (U.S. Food and Drug Administration) as an immunosuppressant in combination with cyclosporine and corticosteroids for prophylaxis of organ rejection; and (c) sirolimus is extensively employed in long-term monotherapy for several diseases, including the treatment of solid organ transplantation [26,27,28], systemic lupus erythematosus [29], autoimmune cytopenia [30], and lymphangioleiomyomatosis [31]. An extended list of diseases that can be treated with sirolimus can be found in Zuccato et al. and Gamberini et al. [19,32]. Moreover, sirolimus and mTOR inhibitors have been evaluated for the possible promotion of health span in adults [33].

Concerning this issue, reduced immune response to the SARS-CoV-2 vaccine has been observed in organ recipients receiving sirolimus-based immunosuppressant therapy [34]. Accordingly, the production of anti-SARS-CoV-2 antibodies in patients receiving sirolimus has been monitored in recent studies performed on liver and kidney transplant recipients [35,36].

Sirolimus is at present employed in two clinical trials conducted on β-thalassemia patients (NCT03877809 and NCT04247750) [32]. These two trials are based on the use of low dosages of sirolimus with the main objective of verifying its efficacy as an in vivo HbF inducer, aiming to reduce the number of transfusions needed with overall good tolerability [19,32]. In this context, for determining the possible effects of sirolimus on anti-SARS-CoV-2 vaccination, β-thalassemia patients participating in the clinical trial NCT04247750 are very informative.

**Figure 1:**
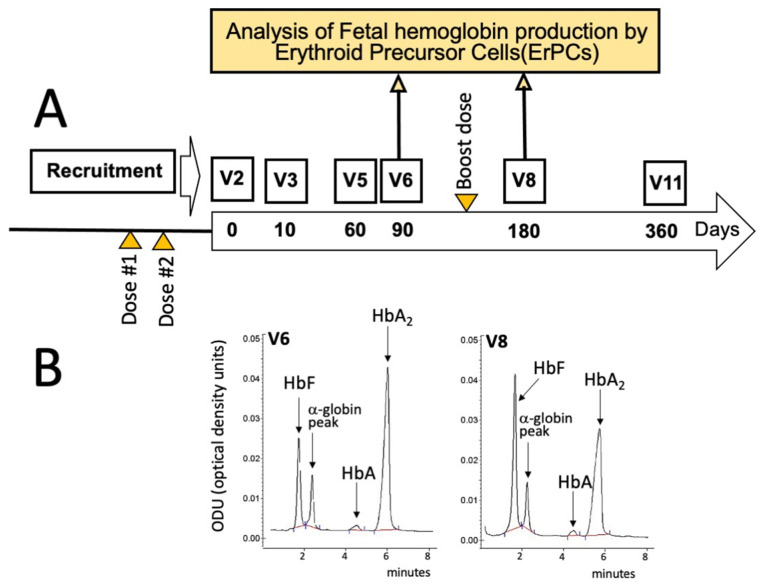
Scheme depicting some key activities within the T04247750 clinical trial (**A**), along with the timing of SARS-CoV-2 vaccination (orange triangles) and blood sampling (yellow arrows) considered in this report. The large longitudinal arrow in panel A identifies the sirolimus treatment that usually started 20 days after the administration of the second dose of the mRNA-1273 (Moderna) vaccine. (**B**) HPLC analysis of the hemoglobins of EPO-cultured ErPCs isolated from the sirolimus-treated patient at V6 (left side of panel (**B**)) and V8 (right side of panel (**B**)) and cultured as described by Zuccato et al. [19] and by Gamberini et al. [32].

**Figure 2:**
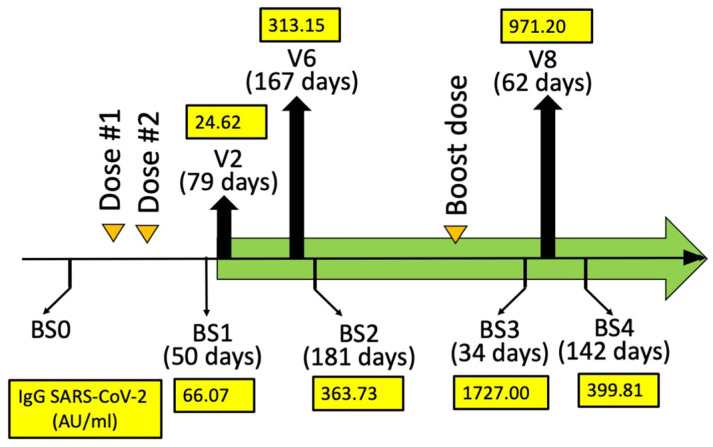
Humoral immune response (AU/mL) quantifying the anti-SARS-CoV-2 IgG at BS1, BS2, BS3, and BS4. The days from mRNA-1273 (Moderna) dose #2 (BS1 and BS2) and booster dose #3 (BS3 and BS4) are indicated in parenthesis. A blood sampling was programmed (BS0) just before the vaccination in order to exclude SARS-CoV-2 infection. Two blood samplings (BS1 and BS2) were programmed after 30–50 (BS1) and 150–200 (BS2) days from BNT162b2 dose #2. Two blood samplings (BS3 and BS4) were programmed after 30–50 (BS3) and 110–160 (BS4) days from BNT162b2 booster dose #3.

According to the protocol summarized in Figure 1A and Figure 2, all the enrolled β-thalassemia patients received two doses of either the BNT162b2 (Pfizer–BioNTech) or the mRNA-1273 (Moderna) vaccines before starting a daily intake of 2 mg of sirolimus as described by Gamberini et al. [32] and by Zuccato et al. [19]. In addition, all the patients received a booster dose about 6 months later (Figure 1A), between V6 and V8.

For the analysis of the effects of sirolimus on parameters of erythropoiesis, visits were programmed after 10 (V3), 90 (V6), 180 (V8), and 360 (V11) days after starting sirolimus intake (Figure 1A). In this report, the HbF production by erythroid precursor cells (ErPCs) isolated from the patient at V6 and V8 and differentiated by culturing with erythropoietin (EPO) was considered. The HbF production by these cells was determined in order to confirm the response of the patient to sirolimus treatment. For determination of anti-SARS-CoV-2 antibodies, blood sampling was performed before the administration of the first dose (BS0), after 30–50 (BS1) and 150–200 (BS2) days from the second dose, then after 30–50 (BS3) and 110–160 days (BS4) from the booster dose (Figure 2). These two blood samplings (early and late) have been programmed, considering several studies indicating that immunity gradually waned after having received the second dose of vaccine [37,38,39].

## 2. Case Presentation

The studied case was a male transfusion-dependent TM (Thalassemia Major) patient, 41 years old, homozygous for the β^0^39-Thalassemia mutation.

He started the first blood transfusion when he was 2 years old, was splenectomized in 1996, and participated in the NCT04247750 clinical trial. More clinical information is reported in Table 1.

According to the general scheme of the study depicted in Figure 1A and Figure 2, he started the intake of sirolimus after about 20 days from the second dose of mRNA-1273 (Moderna) anti-SARS-CoV-2 vaccine. The response of this patient to sirolimus treatment is documented by the HPLC (High-Performance Liquid Chromatography) analysis of the erythropoietin (EPO)-differentiated ErPCs, in agreement with the protocols and the methods described in Gamberini et al. [32] and Zuccato et al. [19].

Figure 1B shows that a sirolimus-mediated increase of HbF was appreciable when EPO-induced ErPCs from the patient treated for 90 days (V6) with sirolimus were compared with ErPCs after 180 days of sirolimus treatment (V8). The HbF content increased from 21.99% (V6) to 40.52% (V8) (1.84-fold). These preliminary analyses support the concept that the patient was responding to sirolimus treatment.

The response to vaccination was determined by quantifying anti-RBD (Spike) antibodies (Abbott Laboratories, Wiesbaden, Germany). The values of the anti-SARS-CoV-2 IgG (immunoglobulin G) (AU/mL) were determined in BS1, BS2, BS3, and BS4 and reported in Figure 2. The highest BS1/BS2 SARS-CoV-2 IgG value of the propositus of this case report study was 362.73. This value is coherent with the data obtained when BS3 and BS4 were considered. In fact, the BS3 SARS-CoV-2 IgG value of the propositus was 1727.00 (Figure 2), which is a value much higher than those found in BS1/BS2. Interestingly, the BS4 value was also high, suggesting that the production of anti-SARS-CoV-2 IgG was durable after more than three months from booster dose #3 of the mRNA-1273 (Moderna) vaccine.

Taken together, these data demonstrated that in this patient, sirolimus allowed the production of SARS-CoV-2 IgGs after vaccination with mRNA-1273 (Moderna) [37].

## 3. Discussion

A reduced immune response to anti-SARS-CoV-2 vaccination has been observed in organ recipients receiving immunosuppressant sirolimus-based treatment [33]. Therefore, there was some concern regarding the fact that monotherapy with sirolimus would reduce the antibody response after SARS-CoV-2 vaccination. On the other hand, recent studies have concluded that negative effects of sirolimus are not present in sirolimus-treated patients [34,35]. For instance, Cheng et al. reported that the neutralizing antibody responses to a SARS-CoV-2 vaccine were unchanged in sirolimus-treated lymphangioleiomyomatosis patients, suggesting that monotherapy with sirolimus or other mTOR inhibitors does not prevent antibody responses to SARS-CoV-2 vaccines [40]. In other studies, a positive, beneficial effect of sirolimus and mTOR inhibitors on anti-SARS-CoV-2 antibody production was reported [24,41,42]. For instance, an immunosuppressive regimen based on low doses of the mTOR inhibitor everolimus was found by de Boer et al. to be associated with a higher humoral response rate after COVID-19 vaccination in elderly kidney transplant recipients [42].

This issue is of relevance for patients with hematological diseases, including β-thalassemia and sickle-cell disease (SCD). In this respect, sirolimus has been proposed as a possible therapeutic strategy for in vivo enhancement of HbF production in β-thalassemia and SCD [8] and is under clinical consideration for β-thalassemia in two trials (NCT03877809 and NCT04247750) [19,33]. However, the immune response of SARS-CoV-2 vaccines was unknown in β-thalassemia patients on monotherapy with sirolimus and other mTOR inhibitors. In this respect, β-thalassemia patients participating in the clinical trial NCT04247750 might be very informative. According to the protocol shown in Figure 1A and Figure 2, all the enrolled β-thalassemia patients received two doses of either the BNT162b2 (Pfizer–BioNTech) or the mRNA-1273 (Moderna) vaccines before starting with the daily intake of 2 mg of sirolimus as described by Gamberini et al. [32] and by Zuccato et al. [19]. The anti-SARS-CoV-2 IgG levels (Figure 2) were found to be comparable to those reported elsewhere of β-thalassemia patients (not treated with sirolimus) [43,44,45].

Our data are also of interest when considered together with our previously published observation on the effects of sirolimus on memory T-cells of sirolimus treated β-thalassemia patients [46]. In this study, Zurlo et al. found that sirolimus treatment has a positive impact on the biological activity and number of memory CD4^+^ and CD8^+^ T cells releasing IFN-γ following stimulation with antigenic stimuli present in immunological memory [46]. As a final comment, we would like to underline that our study might be considered of interest to β-thalassemia and SCD patients living in counties where these diseases are widespread and there is a need to control the COVID-19 pandemic with extensive anti-SARS-CoV-2 vaccination programs.

## 4. Conclusions

The results shown in Figure 2 demonstrate that sirolimus treatment does not prevent the production of anti-SARS-CoV-2 IgG after vaccination with mRNA-1273 (Moderna). In our opinion, this case report study should stimulate further studies on β-thalassemia patients under sirolimus monotherapy. The aim of these further investigations should verify and confirm the safety (or even the positive effects) of sirolimus with respect to the responses to anti-SARS-CoV-2 vaccination. In addition, considering the extensive use of sirolimus for the treatment of other human pathologies (for instance, in organ transplantation, systemic lupus erythematosus, autoimmune cytopenia, and lymphangioleiomyomatosis) [26,27,28,29,30,31], this case report study might be of general interest, as large numbers of patients are currently under sirolimus treatment or will be treated in future clinical trials with sirolimus.

## Figures and Tables

**Table 1:** Clinical parameters and therapies.

Clinical parameters	Comments/ongoing therapies at the time of recruitment to the NCT04247750 trial
A. General parameters	
Genotype	Homozygous for the β^0^39-Thalassemia mutation XmnI polymorphism: -/-
Start of regular transfusion therapy	12 December 1983; age 2.8 years
Transfusion regime	In 2020, 38 units of red blood cells were infused. Mean pre-transfusional Hb: 9.4 g/dL; iron intake: 0.33 mg/kg/die
Start of regular chelation therapy	1 January 1984; age 2.9 years
Chelation therapy	Various schemes were used, including chelating agents in monotherapy or in combination. Since 4 February 2021, alternate combination therapy with desferrioxamine sc (28 mg/kg 3/7) and deferasirox FC per os (20.2 mg/kg 4/7) is ongoing
Iron overload	Severe hepatic and cardiac accumulation was found in 2008 by RM-T2; progressive improvement up to normalization of the deposits was obtained on 6 June 2021 (MRI-T2: cardiac T2 40 ms, LIC 2.19 mg/g liver dry tissue) Serum ferritin: high mean annual values (>2000 ng/mL) from 2008 to 2011; <500 ng/mL from 2019; on 16 March 2021: ferritin 428 ng/mL
Splenectomy	15 March 1996; age 15 years
Adenotonsillectomy	15 September 2008; age 17 years
B. Clinical complications	
Allergic Chronic Asthma (Since pediatric age; allergy developed against alternaria and grasses)	Beclomethasone 200 mcg plus formoterol 2 mcg (Foster^®^): 2 inhalations per day
Piastrinosis (1996, after splenectomy)	Lysine acetylsalicylate (cardirene^®^)300 mg/day
Osteoporosis (2003)	Aledronic acid (dralenos^®^) 70 mg/week
Postpuberal hypogonatropic hypogonadism (2006)	Testosterone gel (tostrex^®^) 40 mg/day (4 pumps in a single dose)
Dilated cardiopathy with ventricular dysfunction secondary to cardiac siderosis (2006)	Bisoprolol 1.25 mg/day, losartan 50 mg plus hydrochlorothiazide 12.5 mg/day
Vitamin D deficiency (2012)	Cholecalciferol (dibase^®^) 1250 IU/day
Growth Hormone deficiency (2014)	Somatotropin (humatrope^®^) 6 mg/day, 6 days/week
Hyper calciuria (2016)	/
Paravertebral ectopic erythropoyesis mass (2017; diameter 2 cm, stable at follow-up)	/
SARS-CoV-2 infection	Never infected

## Data Availability

All of the data produced in the present work are contained in the manuscript. Other information related to the present study is available upon reasonable request to the corresponding authors.
